# Maternal Age and Behavior during Pregnancy Affect the 2D:4D Digit Ratio in Polish Children Aged 6–13 Years

**DOI:** 10.3390/biology11091286

**Published:** 2022-08-30

**Authors:** Aneta Sitek, Iwona Rosset, Magdalena Kobus, Paulina Pruszkowska-Przybylska, Elżbieta Żądzińska

**Affiliations:** 1 Department of Anthropology, University of Lodz, ul. Banacha 12/16, 90-237 Lodz, Poland; 2 School of Medical Sciences, Faculty of Health Sciences, University of Adelaide, Adelaide, SA 5005, Australia

**Keywords:** sex hormones, pregnancy, 2D:4D digit ratio

## Abstract

**Simple Summary:**

The digit ratio (2D:4D) is a marker of prenatal exposure to sex hormones. It is also correlated with many somatic, cognitive, and behavioral traits. The current study examined whether maternal age, health, and lifestyle during pregnancy affect 2D:4D in school-age children of both sexes. The material consisted of digit measurements and questionnaire results for 1219 children (573 boys and 646 girls) aged 6–13 years. The right hand digit ratio in both boys and girls was found to be positively correlated with maternal smoking and negatively with maternal work during pregnancy. Maternal age at childbirth was negatively related to right hand 2D:4D, but only in daughters. These findings suggest that the digit ratio in offspring may be correlated not only with prenatal sex hormone levels, but also with 2D:4D heritability associated with maternal behavior.

**Abstract:**

Background: The length of the second and fourth finger calculated as a ratio (2D:4D) is a marker of prenatal exposure to sex hormones. Higher exposure to testosterone is related to a lower 2D:4D digit ratio, and inversely, higher exposure to estrogen is related to a higher 2D:4D. The digit ratio in humans (prenatally determined by sex hormone levels and androgen receptor activity) is associated with multiple biological, cognitive, and behavioral traits, as well as health in later life. The aim of this study was to verify if maternal traits during pregnancy are associated with 2D:4D among their children. Methods: We investigated 537 boys, 646 girls, and their mothers. The investigation consisted of a questionnaire and a measurement part. In the questionnaire, we included questions about maternal traits during pregnancy such as: illnesses, active and passive smoking, work activity, psychological trauma (death or serious illness of a loved one, divorce, job loss), and age. We performed length measurements of the second and fourth fingers on both hands for both study groups. Results: The GLM analysis showed that children of smoking mothers were characterized by a higher 2D:4D R as compared to their peers whose mothers did not smoke (*β* = 0.10, *p* = 0.0008). In turn, the offspring of women who worked during pregnancy exhibited lower 2D:4D R values than the children of women who did not work (*β* = −0.07, *p* = 0.0233). It should be noted, however, that the effects of those maternal factors were small, as each of them explained less than 1% of 2D:4D R in the population, adjusted for child age and sex. Pearson’s linear correlation revealed that maternal age was negatively correlated with 2D:4D R in daughters (*r* = −0.11, *p* = 0.0137), but not in sons (*r* = 0.02, *p* = 0.6908). The negative correlation of 2D:4D R with maternal age indicates that the daughters of older mothers exhibited lower values of that ratio relative to the same-age daughters of younger mothers. Conclusions: For both study groups, the right-hand digit ratio was positively correlated with maternal smoking and negatively with maternal work during pregnancy. Maternal age during pregnancy was negatively related to right hand 2D:4D only among girls.

## 1. Introduction

The 2D:4D digit ratio is a marker of prenatal proportions of androgens and estrogens [1,2]. This ratio in humans is established within a narrow fetal development window, towards the end of the first trimester of gestation [3,4]. Low 2D:4D values are linked to high prenatal levels of androgens relative to estrogens and vice versa. The digit ratio has also been reported to correlate with the androgen receptor activity in males [5].

However, contrary to this hypothesis, there are rodent and also human studies that have denied that prenatal testosterone affects digit length. Huber et al. [6] presented that the 2D:4D ratio pattern independently of sex among mice is not set up in the prenatal period. Similarly, there are some human studies that did not confirm that fetal and/or maternal sex hormones are associated with the 2D:4D ratio of the offspring [7,8,9,10,11].

Additionally, an allometric hypothesis has also been developed, which indicates that the difference in the 2D:4D ratio is an artifact of the allometric effects of digit growth among both sexes [12]. However, Butovskaya et al. did not confirm that sex differences in 2D:4D are an artifact of allometry [13].

In most populations, males exhibit lower 2D:4D ratios than females [1,14,15,16,17]. Furthermore, child 2D:4D has been shown to be correlated with maternal 2D:4D [18], although some authors have reported this effect only for daughters [11,19].

According to the literature, the digit ratio in humans (prenatally determined by sex hormone levels and androgen receptor activity) is associated with multiple biological, cognitive, and behavioral traits, as well as health in later life. Correlations have been identified between 2D:4D and sperm quality [1], body weight [20], stature [21], body composition [22], skin pigmentation [16,23,24], physical fitness [25], age at menarche [26,27], the risk of autism [28], sexual orientation [29,30], susceptibility to binge drinking [31], dominance [32], thyroid diseases [33], vitamin D and cortisol concentration [34], migraine prevalence [35], hand grip [36], aggressive behavior [37], and even, occupational interests [20,38].

Maternal factors, such as age, health, diet, and lifestyle, significantly affect the quality of the intrauterine environment, and thus influence the fetal phenotype (e.g., birth weight) and biological condition. Those factors can impact the fetus in utero via different signaling pathways, including oxidation, immune/inflammatory, hormonal, and metabolic pathways [39]. As a result, it would be interesting to determine whether maternal factors may affect prenatal androgen and estrogen levels, which are reflected in the offspring’s 2D:4D ratio. The existence of such effects would suggest that prenatal factors may influence human phenotype also by modifying the homeostasis of those hormones.

In the literature, there are few works investigating correlations between maternal factors during prenatal development and the offspring’s 2D:4D ratio. The effect that has garnered the most attention to date is maternal smoking during pregnancy [40].

The objective of the present work is to analyze whether and to what extent maternal factors influencing the intrauterine environment of fetal development (mother’s age, health, and lifestyle) affect the 2D:4D ratio in school-age children.

## 2. Materials and Methods

### 2.1. Participants

The study was conducted in the years 2010–2017 in elementary schools located in Łódź, a major Polish city with 700,000 inhabitants. The inclusion criteria were informed written consent from the parent, oral consent from the child, and questionnaire completion by the parent.

### 2.2. Procedure

The questionnaire collected information about the pregnancy with the child under study, with items asking about the use of medications for high-risk pregnancy, maternal illnesses during pregnancy, maternal active and passive smoking during pregnancy, maternal work during pregnancy, and maternal psychological trauma during pregnancy (death or serious illness of a loved one, divorce, job loss), as well as mother’s and child’s birth dates (to calculate maternal age at childbirth and child age at examination).

Every participating child had the length of their second (2D) and fourth (4D) fingers measured between the *pseudophalangion* and *dactylion*, both in the left and right hands, by means of a sliding compass with an accuracy of 1 mm. Those measurements were used for calculating 2D:4D digit ratios for the right and left hands.

The study material consisted of measurements and questionnaires conducted for a total of 1219 children (573 boys and 646 girls) aged 6–13 years. The sample size varied between the various analyses due to missing data in some questionnaires. Incomplete records were not rejected, as that would have considerably reduced the dataset (complete records were obtained for 897 children, or 73.6% of the participants).

### 2.3. Ethical Considerations

The study was approved by the Bioethics Board of the University of Lodz (KBB-UŁ/II/11/2010). We confirmed that all research was performed in accordance with relevant guidelines/regulations. We obtained the informed written consent of the parent of each examined child.

### 2.4. Statistical Analysis

The distribution of all continuous variables was normal and was examined using the Shapiro–Wilk test; thus, parametric tests were applied in the further analyses.

The left and right 2D:4D ratios and maternal age at childbirth for boys and girls were compared with Student’s *t*-test for equal or unequal variances, depending on the *F* test. Covariances between the left and right 2D:4D ratios, between the digit ratio and the child’s chronological age, and between the child’s digit ratio and maternal age were described by means of Pearson’s linear correlation coefficients. In each case, the choice of linear correlation was dictated by the graphical interpretation of a scatter plot of the selected variables.

The frequencies of categorical variables in the groups of boys and girls were compared using the chi-squared test (χ^2^) with Yates correction (the correction was necessary because each time, 2 × 2 contingency tables were analyzed).

Correlations between the various prenatal factors and the left and right hand digit ratios were evaluated using the generalized linear model (GLM), which was also applied to estimate the effects of interactions between the analyzed factors and child sex on left and right 2D:4D. The effect size for a given factor or interaction was assessed by calculating omega squared (*ω*^2^), estimating the proportion of variance attributable to a given variable in the entire population. All analyses were implemented in the Statistica PL ver. 13 (TIBCO Software, Palo Alto, CA, USA).

## 3. Results

The correlation coefficient between the right and left 2D:4D ratios for both groups of children (both boys and girls) was *r* = 0.63. According to the literature, the coefficient should be between 0.50 and 0.70 [14], and so, the presented measurements should be deemed accurate.

Statistical analysis revealed that both the right hand digit ratio (2D:4D R) and the left hand digit ratio (2D:4D L) in girls were higher than the corresponding ratios in boys, which shows that the mean relative length of the index finger is greater in females (Table 1). While 2D:4D R was not correlated with age in either sex, 2D:4D L was positively correlated with age at examination in girls, but not in boys (Table 2). This means that in older girls, 2D:4D L was higher (more feminine) than in younger girls. However, the strength of that correlation was very low (*r* = 0.09).

No significant differences in maternal prenatal factors were found between the groups of boys and girls (Table 3), which means that none of them affected either sex to a greater extent (or more frequently).

The main objective of the statistical analysis was the evaluation of the correlations between maternal prenatal factors and children’s left and right hand digit ratios. Due to the fact that 2D:4D R and 2D:4D L differed between boys and girls, calculations were adjusted for sex. Furthermore, despite the fact that children’s age was correlated with the digit ratio for only one hand and only in girls (Table 2), both 2D:4D R and 2D:4D L were adjusted for age, for the sake of consistency and the clarity of interpretation.

**Figure 1:**
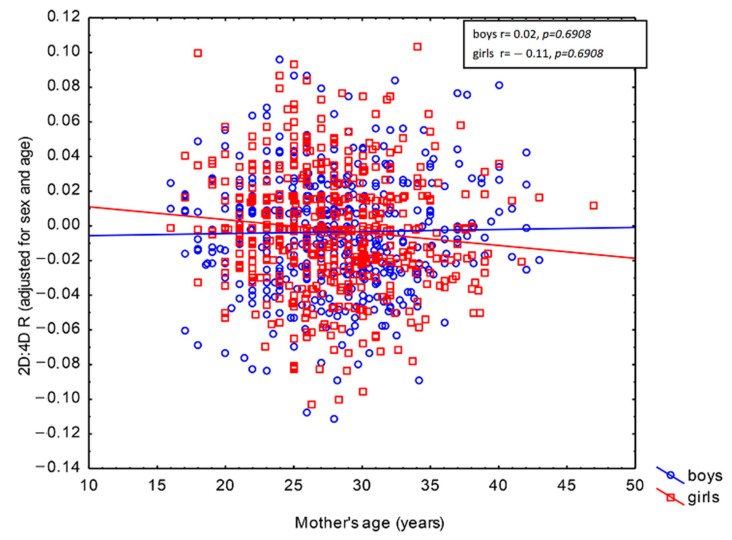
Correlation between maternal age at childbirth and the right 2D:4D digit ratio in children aged 6–13 years depending on sex.

The GLM revealed correlations between 2D:4D R and maternal active smoking and work during pregnancy (Table 4). The children of smoking mothers were characterized by higher 2D:4D R as compared to their peers whose mothers did not smoke (*β* = 0.10, *p* = 0.0008). In turn, the offspring of women who worked during pregnancy exhibited lower 2D:4D R values than the children of women who did not work (*β* = −0.07, *p* = 0.0233). It should be noted, however, that the effects of those maternal factors were small, as each of them explained less than 1% of 2D:4D R in the population, adjusted for child age and sex (*ω*^2^ = 0.84% for maternal active smoking and *ω*^2^ = 0.34% for maternal work). Both of these correlations held irrespective of the child’s sex (*p* = 0.8143 for maternal active smoking × child sex interaction and *p* = 0.7696 for maternal work × child sex interaction). This shows that the direction of these prenatal effects on 2D:4D R was the same in boys and girls (Table 4). Maternal age at childbirth was not correlated with 2D:4D R adjusted for child age and sex in the studied population (*β* = −0.05, *p* = 0.1410). However, its effect was found to depend on child sex (*p* = 0.0421 for maternal age at childbirth × child sex interaction; see Table 4). Furthermore, the population effect of this interaction was very weak (*ω*^2^ = 0.32%). Pearson’s linear correlation revealed that maternal age was negatively correlated with 2D:4D R in daughters (*r* = -0.11, *p* = 0.0137), but not in sons (*r* = 0.02, *p* = 0.6908). The negative correlation of 2D:4D R with maternal age indicates that the daughters of older mothers exhibited lower values of that ratio relative to the same-age daughters of younger mothers (Figure 1).

None of the analyzed prenatal factors were found to modify the left 2D:4D ratio, either directly or in interaction with child sex (Table 5).

## 4. Discussion

The present study revealed lower right and left hand digit ratios in male vs. female children, corroborating the sexually dimorphic pattern described by many other authors [18,32]. However, there are some studies that question these findings [6,7,8,9,10,11]. Furthermore, it was also shown that 2D:4D R did not depend on the age of schoolchildren (6–13 years). In the case of 2D:4D L, a positive, but very weak (*r* = 0.09) correlation with age was identified only for girls. This is in line with many other studies suggesting that the digit ratio is established in utero and remains essentially unchanged throughout subsequent ontogenesis [1,23,24,41]. However, some papers have suggested that the digit ratio increases with age [14,15,18]. This disparity of findings might be attributable to different study populations, a low test power giving rise to false positive or false negative results, or excessively narrow or diverging age groups [17].

There are few reports in the literature on the relationship between prenatal variables and children’s digit ratio. The current study showed that some maternal factors (mother’s age, smoking, and work during pregnancy) were correlated with 2D:4D R in school-age offspring. Valez et al. did not find any effects of maternal periconceptional smoking (one year before through the first trimester of pregnancy) on right and left hand 2D:4D in 2–5-year-old girls and boys [18]. In turn, Rizwan, Manning, and Brabin [40] reported that school-age boys whose mothers smoked during pregnancy had lower 2D:4D R as compared to their peers born to non-smoking mothers. The difference remained significant after controlling for the effects of age, height, weight, and birth weight. Other household smoking patterns (e.g., paternal smoking during pregnancy and maternal or paternal smoking outside pregnancy) were not associated with male offspring’s 2D:4D. Female offspring’ 2D:4D was unaffected by maternal smoking [40]. In addition, Rizwan, Manning, and Brabin noted that their findings were consistent with the studies by Kandel and Udry [41] and Sowers et al. [42], who found that testosterone levels and smoking are positively correlated in pregnant women. Furthermore, according to Kitawaki et al. [43], maternal smoking during pregnancy decreases the levels of cytochrome P450 aromatase in the human placenta, reducing its ability to produce estrogen. Given the above, the higher 2D:4D R in the offspring of mothers smoking during pregnancy found in this study seems surprising (a positive correlation between smoking mothers and 2D:4D R in children occurred in both boys and girls, as indicated by the non-significant maternal smoking×child sex interaction). On the other hand, this result appears to be in line with the study encompassing a large international sample conducted by Manning and Fink [44], who indicated that maternal smoking is associated with their high 2D:4D ratios. Thus, the present findings may be attributable to the positive correlation between the digit ratios of mothers and children reported by Velez et al. [18]. This would mean that the offspring of smoking mothers exhibit higher 2D:4D via an association with the maternal digit ratio rather than because of altered sex hormone homeostasis during prenatal development. Positive effects of smoking on testosterone are likely not via any direct action on the male fetal gonads, supporting firstly the fact that female and male fetuses appear to be equally affected and that the influence is not via a direct effect. While maternal smoking has a positive impact on mid-pregnancy amniotic testosterone levels for fetal males, it has no effect on the equivalent levels of the fetal testis hormone INSL3 [45]. Further research is needed to investigate this effect. Unfortunately, this study did not control for maternal 2D:4D, and so, it is impossible to unequivocally determine the underlying cause of the observed phenomenon.

Another correlation identified in the current study involved maternal work during pregnancy, which was linked to lower right hand digit ratios in offspring of both sexes as compared to the children of non-working mothers. In the absence of any literature reports on the subject, it may be hypothesized that this is again attributable to maternal behavior and 2D:4D heritability. In a number of studies, it has been shown that behavioral traits are correlated with the digit ratio both in females and males. In particular, it has been found that lower digit ratios in both sexes are related to higher risk taking [46], higher aggression [16,47], and higher sensation-seeking [16]. It has also been established that women with lower 2D:4D ratios are more assertive and competitive [48], more willing to engage in high-risk sports (e.g., judo and boxing) [49], and more likely to join the uniformed services (e.g., police force) [20]. In light of these psychological and behavioral propensities, it may be expected that women with lower digit ratios are also more likely to remain professionally active during pregnancy than those with higher (more feminine) 2D:4D. Consequently, in light of digit ratio heritability, the offspring of such women (both sons and daughters) may also exhibit lower 2D:4D. This hypothesis should be tested in future investigations.

The last effect identified in this study was a negative correlation between maternal age at childbirth and 2D:4D in girls, which means that the daughters, but not sons, of older mothers tended to have a lower (more masculine) right hand digit ratio. According to the literature data, plasma testosterone levels in women are negatively associated with age [19] (which would suggest a positive correlation between maternal age at childbirth and offspring’s 2D:4D). However, that does not necessarily imply that there exists an association between the age of pregnant women and amniotic testosterone levels [19] The same authors reported that 2D:4D variability in female newborns was affected to similar degrees by maternal heritability and testosterone levels in the amniotic fluid, which is mostly generated by the fetus, rather than the mother. Thus, it appears that the results presented in this study do not reflect prenatal sex hormone levels, but rather arise from mother–daughter digit ratio heritability. In women, 2D:4D has been reported to be positively correlated with reproductive success measured by the number of offspring [32,50], as well as with its correlates such as: preferred age at first child, frequency of sex, strength of sex drive [32], age at birth of the last child, and the length of the reproductive lifespan [50]. In light of these findings, it seems likely that women with a lower (more masculine) digit ratio become mothers at a later age and their offspring tend to inherit lower 2D:4D, which would explain the observed effect. However, this hypothesis should be confirmed in a future study specially designed to address this issue. Although maternal heritability of 2D:4D has been also reported for children of both sexes [18], according to other authors, mother’s 2D:4D is correlated only with the digit ratio of daughters [17,19]. Ventura et al. suggested that the lack of 2D:4D correlation between mothers and sons is attributable to the fact that high prenatal testosterone concentrations may induce a level of individual variability capable of breaking the correlation between sons’ and mothers’ digit ratios [19], which would account for the absence of such an effect also in this study.

It should be noted that our study has some limitations. The strength of each of the identified correlations was small, accounting for less than 1% of right hand digit ratio variation adjusted for child age and sex. Thus, maternal prenatal factors appear to modify offspring’s digit ratio only to a small degree. Finally, all of the identified correlations involved children’s right hand digit ratio, which is consistent with many other publications [1,15,24,47,51]. However, there is a simultaneously limitation of this study. When the effect was small (<1%) and was not observed on both hands, this may be an artifact of this study. Additionally, limitations include the declarative nature of information about maternal prenatal factors and the lack of data on maternal 2D:4D ratios. Finally, the advantage of this study is the large sample of children of both sexes and different ages, which authenticated the small maternal effect that we pointed out.

## 5. Conclusions

In summary, this study suggested that some maternal prenatal factors might influence offspring’ digit ratio. These correlations are probably attributable to 2D:4D heritability associated with maternal behavior. The paper presents new data concerning 2D:4D determinants that were not previously analyzed in the context of 2D:4D variation in offspring. We showed that in both groups of children, the right-hand digit ratio was positively correlated with maternal smoking and negatively with maternal work during pregnancy. Maternal age during pregnancy was negatively related to right hand 2D:4D only among girls.

## Figures and Tables

**Table 1:** Comparison of right and left hand digit ratios in children, by sex.

Digit Ratio	Boys *n* = 537		Girls *n* = 646		*p*
	$\bar{x}$	SD	$\bar{x}$	SD	
2D:4D R	0.9740	0.0336	0.9842	0.0323	<0.0001
2D:4D L	0.9725	0.0309	0.9802	0.0306	<0.0001

*n*—sample size, $\bar{x}$ —arithmetic mean, SD—standard deviation, *p*—probability for Student’s t-test.

**Table 2:** Evaluation of correlations between right and left hand digit ratios in children and their chronological age.

Correlation	Boys *n* = 537		Girls *n* = 646	
	*r*	*p*	*r*	*p*
2D:4D R × child age	0.08	0.0590	0.01	0.7759
2D:4D L × child age	0.08	0.0625	0.09	0.0185

*r*—Pearson’s correlation coefficient, *p*—probability for Student’s *t*-test of the null hypothesis such that *r* = 0.

**Table 3:** Maternal prenatal factors by child sex.

Maternal Prenatal Factors		Total	Boys	Girls	*p*
Maternal age at childbirth	$\bar{x}$	27.97	28.05	27.89	0.6311
	SD	4.97	5.16	4.81	
	*n*	990	462	528	
Use of medications for high-risk pregnancy	Yes	407	191	216	0.9780
	No	808	380	428	
	Total	1215	571	644	
Maternal illness during pregnancy	Yes	188	83	105	0.4310
	No	993	474	522	
	Total	1181	554	627	
Maternal active smoking during pregnancy	Yes	138	65	73	0.9469
	No	1081	508	573	
	Total	1219	573	646	
Maternal passive smoking during pregnancy	Yes	422	196	226	0.8549
	No	780	368	412	
	Total	1202	564	638	
Maternal work during pregnancy	Yes	575	272	303	0.8295
	No	632	294	338	
	Total	1207	566	641	
Maternal psychological trauma during pregnancy	Yes	103	42	61	0.2315
	No	1102	523	579	
	Total	1205	565	640	

*p*—probability (Student’s *t*-test for maternal age, *χ*^2^ with Yates’s correction for the other variables).

**Table 4:** Correlations between maternal prenatal factors and the right hand 2D:4D digit ratio ^1^ in children aged 6–13 years (GLM).

Prenatal Factors (Independent Variables)	Direct Effect of Prenatal Factor				Prenatal Factor × Child Sex Interaction		
	*β*	*t*	*p*	*ω*^2^ (%)	*F*	*p*	*ω*^2^ (%)
Maternal age at childbirth	−0.05	−1.47	0.1410	0.12	4.14	0.0421	0.32
Use of medications for high-risk pregnancy—Yes vs. No	−0.01	−0.37	0.7149	0.00	0.05	0.8147	0.00
Maternal illness during pregnancy—Yes vs. No	0.01	0.35	0.7250	0.00	0.03	0.8643	0.00
Maternal active smoking during pregnancy—Yes vs. No	0.10	3.36	0.0008	0.84	0.06	0.8143	0.00
Maternal passive smoking during pregnancy—Yes vs. No	0.04	1.40	0.1628	0.08	0,09	0.7607	0.00
Maternal work during pregnancy—Yes vs. No	−0.07	−2.27	0.0233	0.34	0.09	0.7696	0.00
Maternal psychological trauma during pregnancy—Yes vs. No	0.03	1.07	0.2860	0.01	0.41	0.5220	0.00

^1^ The 2D:4D R adjusted for child sex and chronological age. *Β*—standardized linear regression coefficient. *t*—test evaluating the significance of the linear regression coefficient. *F*—test evaluating the ratio of the variance explained by a given effect or interaction to the variance of the error. *p*—probability. *ω*^2^—estimator of the variance of the dependent variable explained by the independent variable/interaction in the entire population. GLM—generalized linear model.

**Table 5:** Correlations between maternal prenatal factors and the left hand 2D:4D digit ratio ^1^ in children aged 6–13 years (GLM).

Prenatal Factors (Independent Variables)	Direct Effect of Prenatal Factor				Prenatal Factor × Child Sex Interaction		
	*β*	*t*	*p*	*ω*^2^ (%)	*F*	*p*	*ω*^2^ (%)
Maternal age at childbirth	−0.04	−1.30	0.1951	0.07	0.75	0.3855	0.00
Use of medications for high-risk pregnancy—Yes vs. No	−0.02	−0.81	0.4208	0.00	0.31	0.5749	0.00
Maternal illness during pregnancy—Yes vs. No	0.01	0.37	0.7111	0.00	0.14	0.7105	0.00
Maternal active smoking during pregnancy—Yes vs. No	0.04	1.22	0.2216	0.04	0.52	0.4702	0.00
Maternal passive smoking during pregnancy—Yes vs. No	−0.01	−0.20	0.8437	0.00	0.03	0.8726	0.00
Maternal work during pregnancy—Yes vs. No	−0.02	−0.67	0.5053	0.00	0.17	0.6838	0.00
Maternal psychological trauma during pregnancy—Yes vs. No	−0.01	−0.42	0.6743	0.00	1.66	0.1982	0.05

^1^ The 2D:4D R adjusted for child sex and chronological age. *Β*—standardized linear regression coefficient. *t*—test evaluating the significance of the linear regression coefficient. *F*—test evaluating the ratio of the variance explained by a given effect or interaction to the variance of the error. *p*—probability. *ω*^2^—estimator of the variance of the dependent variable explained by the independent variable/interaction in the entire population. GLM—generalized linear model.

## Data Availability

The datasets generated and/or analyzed during the current study are available from the corresponding author upon reasonable request.
